# Low levels of awareness and motivation towards cancer prevention amongst the general public in Sweden: a cross-sectional study focusing on the European Code Against Cancer

**DOI:** 10.1186/s12889-025-22803-3

**Published:** 2025-05-07

**Authors:** Cecilia Hultstrand, Ellen Brynskog, Andreas Karlsson Rosenblad, Anna-Lena Sunesson, Thomas Björk-Eriksson, Lena Sharp

**Affiliations:** 1 Regional Cancer Centre North. Norrlands universitetssjukhus, Umeå, 901 85 Sweden; 2 Department of nursing, Umeå University, Umeå, 901 87 Sweden; 3 Regional Cancer Centre West. Medicinaregatan 18G, Gothenburg, 413 90 Sweden; 4 Department of health sciences, Karlstad University, Karlstad, 651 88 Sweden; 5 Regional Cancer Centre Stockholm-Gotland, Lindhagensgatan 98, Stockholm, 112 18 Sweden; 6 Department of Statistics, Uppsala University, Uppsala, Sweden; 7 Department of Diagnostics and Intervention, Umeå University, Umeå, 901 87 Sweden; 8 Department of Oncology, Institute of Clinical Sciences, Sahlgrenska Academy, University of Gothenburg, Gothenburg, Sweden

**Keywords:** European code against Cancer, Cancer prevention, Health literacy

## Abstract

**Background:**

The European Code Against Cancer (ECAC) aims to increase the awareness of modifiable cancer risk factors among the general public. A goal set by the European Commission was that 80% of European citizens should be aware of this code by 2025. This study aims to examine the awareness and attitudes towards the ECAC among the general public in Sweden.

**Methods:**

A randomly selected sample of 1520 Swedes (18–84 years old) were recruited from a survey panel and invited to respond to an online study-specific questionnaire. The questionnaire included general questions regarding cancer prevention, as well as awareness and attitudes specific to the ECAC. Data were analysed univariately and with adjusted logistic regression, using post-stratification weights based on gender, age, education, and expressed political party orientation.

**Results:**

In total, 3.7% of the respondents had heard about the ECAC before taking this survey. Respondents with a college/university education were more likely to have heard about the ECAC (odds ratio [OR] 2.23; 95% confidence interval [CI] 1.23–4.06). Males (OR 0.56; 95% CI 0.32–0.99), and those living alone (OR 0.47; 95% CI 0.23–0.95) were less likely to have heard about the ECAC. In total, 60.6% of the respondents agreed with the ECAC recommendations, while 27.4% reported that their motivation to improve their lifestyle increased after reading the ECAC.

**Conclusions:**

Awareness of the ECAC among the general public in Sweden is very low. Still, a majority seem to agree with its recommendations. The results also indicate that the ECAC motivates some, but far from all, to improve their lifestyle habits to reduce their cancer risk. Consequently, further research is warranted on how the ECAC best could and should be used in order to improve cancer prevention awareness and motivation.

**Supplementary Information:**

The online version contains supplementary material available at 10.1186/s12889-025-22803-3.

## Background

The cancer burden in Europe is extensive. Even though cancer mortality rates are decreasing in many countries, both cancer incidence and prevalence are increasing. In 2020, the risk of being diagnosed with cancer among people aged < 75 years was 31% among men and 24% among women [1]. In 2022, one European citizen was diagnosed with cancer every 11th second. In less than two decades, the cancer incidence is expected to increase with 18% [2]. However, it is estimated that approximately 40% of cancer cases within the European Union (EU) could be prevented if exposures to known risk factors are reduced and effective prevention strategies are implemented [3]. Globally, 44% of all cancer deaths during 2019 were reported to be associated with modifiable risk factors. Behavioural risks constituted the largest attributable burden [4]. Thus, stronger commitment to cancer prevention, including raising the awareness of cancer risk factors, is prioritised in Europe’s Beating Cancer plan [3]. In Europe, the three most common cancer types are breast, colorectal, and lung cancer [1]. These cancer types are all associated with modifiable risk factors, such as tobacco and alcohol consumption, diet, and physical activity [5].

Furthermore, there is a socioeconomic gradient in all aspects of the cancer continuum, including cancer prevention. Research indicates that socioeconomic inequities influence exposure to cancer risk factors, as well as access to screening and other preventive services [6–8]. Research has shown that people with lower education and/or income levels tend to be more exposed to some cancer risk factors (e.g. tobacco, obesity, physical inactivity) compared to people with higher education and income levels [2]. Effective preventive strategies have great potential to decrease these inequities [9, 10] and are paramount in tackling the cancer burden.

## The European Code Against Cancer

The European Commission initiated and presented the first version of the European Code Against Cancer (ECAC) in 1987. The ECAC aims to increase the awareness of modifiable risk factors for cancer in the general population. In 2014, the current 4th edition of the ECAC was published (Table 1). The latest revision was led by the International Agency for Research on Cancer (IARC). The aim was to produce a communicable set of evidence-based cancer prevention recommendations, suitable for a broad target audience, focusing on the actions that individuals could take to reduce their cancer risk [11–13].

The ECAC consists of 12 recommendations (Table 1). These recommendations, together with more in-depth information as well as infographics are published in 23 languages on an IARC website [11]. The code has been promoted by both public and non-governmental stakeholders across Europe [14]. The ECAC is currently under revision, and a fifth version is expected to be published during 2025.

**Table 1:** The European CodeAgainst Cancer (ECAC), 4th edition

1.	Do not smoke. Do not use any form of tobacco.
2.	Make your home smoke free. Support smoke-free policies in your workplace.
3.	Take action to be a healthy body weight.
4.	Be physically active in everyday life. Limit the time you spend sitting.
5.	Have a healthy diet:
	• Eat plenty of whole grains, pulses, vegetables and fruits.
	• Limit high-calorie foods (foods high in sugar or fat) and avoid sugary drinks.
	• Avoid processed meat; limit red meat and foods high in salt.
6.	If you drink alcohol of any type, limit your intake. Not drinking alcohol is better for cancer prevention.
7.	Avoid too much sun, especially for children. Use sun protection. Do not use sunbeds.
8.	In the workplace, protect yourself against cancer-causing substances by following health and safety instructions.
9.	Find out if you are exposed to radiation from naturally high radon levels in your home. Take action to reduce high radon levels.
10.	For women:
	• Breastfeeding reduces the mother’s cancer risk. If you can, breastfeed your baby.
	• Hormone replacement therapy (HRT) increases the risk of certain cancers.
	• Limit use of HRT.
11.	Ensure your children take part in vaccination programmes for:
	• Hepatitis B (for newborns).
	• Human papillomavirus (HPV) (for girls).
12.	Take part in organised cancer screening programmes for:
	• Bowel cancer (men and women).
	• Breast cancer (women).
	• Cervical cancer (women).

Sources: International Agency for Research on Cancer, World Health Organization.

Information and communication campaigns to raise awareness among the general public, is a prevention strategy utilized worldwide in a variety of health promotion contexts. The rationale is that people have a right to health information and that increased awareness and/or knowledge can empower people, leading to improved individual health behaviour [15, 16]. Europe’s Beating Cancer plan aims to make at least 80% of the population aware of the ECAC by 2025 [3]. In Sweden and elsewhere, scholars have explored awareness related to cancer risk factors and symptoms [17–21]. However, to our knowledge, awareness of the ECAC among the general public in Sweden has not previously been studied.

## Aim

The main aim of this study is to examine the awareness of and attitudes towards the ECAC among the general public in Sweden. Furthermore, the study aims to investigate attitudes towards cancer prevention in general among this population, as well as to examine possible differences between population groups. The study is also intended to function as a cohort profile description, providing details regarding the design and methodological aspects, as a reference for future studies using the same data.

## Methods and material

### Study design

A cross-sectional study design, utilizing an online questionnaire, was used to measure awareness of and attitudes towards the ECAC among the general public in Sweden. This study is part of the Joint Action Prevent Non-Communicable Diseases and Cancer (EU JA Prevent NCD).

### Participants

Participants were recruited from *Sverigepanelen* (the Sweden Panel), an online survey panel operated by the data analysis company Novus [22]. The panel is randomly recruited, closed (i.e. it is not possible to self-register to be included in the panel) and includes approximately 50,000 panellists aged 18–84 years living in Sweden. A sample of 1520 panellists was randomly recruited by email to participate in the present study.

### Data collection

Data were collected during the period April 2–11, 2024. The first 100 responses were used as a pilot test and were screened for missing data and for how well the respondents matched the intended population. No issues were identified at this point, and the data collection continued. In total, 3099 panellists were consecutively recruited, until the intended goal of 1520 panellists had accepted to participate and completed the questionnaire (corresponding to a response rate of 49.0%). The sample size was chosen in order to enable valid sub-analyses. The inclusion criteria were participating in the online survey panel and consenting to participate. According to Novus’ protocol, panel members were not eligible for participation if they had submitted a response to a survey with a related topic, such as health or lifestyle, during the past six months (in order to not fatigue panel members). To adjust for possible biases in the sample compared to the target population, the sample was post-stratified with regards to gender, age, education, and expressed political party orientation.

### Questionnaire

A study-specific online questionnaire (developed by the research team) was used. Most questions were adopted and adapted from previous studies by Ritchie et al., [23], Keeney et al., [24], and from The Cancer Awareness Measures plus (Cancer research UK) [25]. The questionnaire included 15 questions on awareness of risk factors for cancer, ECAC awareness, and attitudes and behaviours related to cancer prevention. A translated English version of the questionnaire is presented in Supplementary material 1. The ECAC was presented to participants in the questionnaire, followed by question on awareness; “Had you heard about the European Code Against Cancer before taking part in this survey?”. All risk factors mentioned in question # 3 were presented to the participants in random order, as were questions # 4–6, 11–14, and all objects in question # 15. It was not possible to change registered responses. The questionnaire was programmed and administrated to the panel by Novus.

In addition to the questionnaire, data on age, gender, personal income, education level, geographical area, marital status, urban/rural living, country of birth, and parents’ country of birth were collected for each participant from Novus’ data base. These data are updated by panellists approximately every six months.

### Study variables

#### Outcomes

The respondents’ awareness of the ECAC was measured by the following questions:

1. “Had you heard about the European Code Against Cancer before taking part in this survey?” (question # 7).
2. ”To what extent do you agree with the following statements?”
  - “I have learned something new about cancer prevention after reading the European Code Against Cancer.” (question # 9).
  - “I agree with the recommendations described in the European Code Against Cancer.” (question # 10).

Question 1 had the following response options; “Yes”, “No”, “Don’t know”, which were dichotomized as “Yes” and “No”, with “Don’t know” responses included in the “No” category. Questions 2a and 2b had Likert scale response options (1–5 points), with 1 point labelled as “Does not agree at all” and 5 points as “Totally agree”, in addition to the “Don’t know” option. For these two questions, responses at 4–5 points were categorized as “Yes”, while all other answers were categorized as “No”.

Attitudes and behaviours related to lifestyle factors and cancer prevention, were measured by the following questions:

3. ”To what extent do you agree with the following statements?”
  - “My motivation to improve my lifestyle has increased after reading the European Code Against Cancer.” (question # 11).
  - “Information about cancer prevention has made me change my lifestyle.” (question # 12).
  - “I intend to change my lifestyle to reduce my risk of cancer.” (question # 13).

The response options were identical to questions 2a and 2b and were dichotomized in the same way.

#### Predictors

Regarding demographic characteristics, gender had the response options “Male”, “Female”, and “Other”. However, none of the respondents chose “Other”. Therefore, it was used as a binary variable (“Male”/Female”). Age (years) was provided in discrete form and categorized as 18–34, 35–49, 50–64, or 65–84 years old. National background was categorized as Swedish if the respondent and at least one parent was reported as having been born in Sweden and otherwise as foreign or missing (if no information about the respondent’s country of birth was provided). Education was reported as “Primary school” (*Grundskola eller motsvarande*), “Secondary school” (*Gymnasium eller motsvarande*), “College/university” (*Universitet/högskola*), or “None completed”. No one responded “None completed”, therefore education was used as a three-level categorical variable (primary school, secondary school, college/university). Personal income (Swedish Krona (SEK)/month) was provided as income brackets of 10,000 SEK/month, with < 10,000 SEK/month as the lowest and ≥ 70,000 SEK/month as the highest income bracket (10,000 SEK ≈ €885). In addition, the following response options were included: “Does not want to disclose”, “Don’t know”, and “No income”. These were categorized into four categories as “< 20,000 SEK/month” (including “no income”), “20,000–39,999 SEK/month”, “≥ 40,000 SEK/month”, and “Don’t know/Does not want to disclose”. Marital status was provided as “Married”, “Cohabiting”, “Living alone”, “Partnership” “Living with parents, or “Other”. For the purpose of this study, the responses were categorized as living alone (yes/no), with those reporting their marital status as “living alone” categorized as “Yes” and all other categorized as “No”. Finally, geographic area was categorised into the six Swedish health care regions (HCRs): “Stockholm-Gotland”, “Mid-Sweden”, “South East”, “South”, “West”, and “North”.

### Statistical analyses

Categorical data are presented as frequencies and percentages, *n* (%), while discrete and continuous data are provided as mean values with accompanying standard deviations (SDs). In the statistical analyses, national background was dichotomized as Swedish/other and education level as college/university education (yes/no), while income (SEK/month) was categorized into three levels: < 20,000 SEK/month (including “No income”) or “Other” (including “Don’t know” and “Does not want to disclose”), 20,000–39,999 SEK/month, and ≥ 40,000 SEK/month. All statistical analyses utilized the post-stratified weights, using the R package survey version 4.4 [26]. Tests of differences were for categorical data performed using Pearson’s χ^2^-test applying the Rao-Scott second-order correction, with *P*-values computed using a Satterthwaite approximation to the distribution and denominator degrees of freedom as given by Thomas and Rao [27]. To estimate the magnitude of the association between demographic characteristics (predictors) and the six variables used to measure awareness of ECAC and attitudes and behaviours related to lifestyle factors and cancer prevention (outcomes), weighted adjusted logistic regression models were calculated using generalised linear models with a quasi-binomial family and a logit link function together with inverse-probability weighting and design-based standard errors. All demographic variables were used simultaneous as predictors (independent variables), with age (years) included as a continuous variable, “Female” used as reference category for gender, “Other” as reference category for national background, “No” as reference category for college/university education, “< 20,000 / Other” as reference category for income, “No” as reference category for living alone, and “Stockholm-Gotland” as reference category for HCR.

All statistical analyses were performed using R 4.3.1 (R Foundation for Statistical Computing, Vienna, Austria) with two-sided *P*-values < 0.05 considered statistically significant.

## Results

No panellists were excluded from being invited to the study for having responded to a survey in a similar field of inquiry during the past six months. The weighted and unweighted distribution of the demographic characteristics among the 1520 respondents are presented in Table 2. For gender, there was a slight predominance of men among the respondents (50.5% compared to 49.5%), with the difference in frequency between the weighted and unweighted samples being negligible. Neither of the genders thus tending to be over- or underrepresented, compared to the underlying general population. Respondents aged ≥ 50 years old tended to be overrepresented, resulting in responses from this group being down-weighted and the responses for those aged < 50 years old, in turn being up-weighted. In particular, the age group 18–34 years was underrepresented, with only 288 (18.9%) of the respondents belonging to this group, which was then upweighted to 428 (28.1%) of the respondents. The overall unweighted mean (SD) age was 53.3 (17.5) years, while the weighted mean (SD) age was 48.7 (18.5) years.

In total, 1243 (81.8%) reported having a Swedish national background, which was only a slight overrepresentation, resulting in a down-weight to 1216 (80.0%) of the respondents. College/university educated individuals were heavily over-represented, with a total of 949 (62.4%) of the respondents, thus also being heavily down-weighted to 594 (39.1%), while those having a secondary school education level were up-weighted from 506 (33.3%) to 812 (53.4%). Income was, on the other hand quite representative, with a slight up-weighting of those earning < 20,000 SEK/month from 323 (21.2%) to 427 (28.1%), while the group earning ≥ 40,000 SEK/month was down-weighted from 538 (35.4%) to 433 (28.5%). About one in five (*n* = 366; 24.1%) were living alone and a similar proportion (*n* = 393; 25.9%) were living in Stockholm-Gotland, the largest of the six HCRs. For both variables, the number of respondents in each category was quite representative, resulting in only minor up- and down-weightings.

**Table 2:** Weighted and unweighted distribution of demographic characteristics among the 1520 respondents

	Unweighted		Weighted	
Variable	*n*	%	*n*	%
Gender				
• Male	767	50.5	768	50.5
• Female	753	49.5	752	49.5
Age (years)^a^				
• 18–34	288	18.9	428	28.1
• 35–49	362	23.8	376	24.7
• 50–64	407	26.8	361	23.8
• 65–84	463	30.5	355	23.4
National background				
• Swedish	1243	81.8	1216	80.0
• Foreign	102	6.7	99	6.5
• Missing	175	11.5	205	13.5
Education level				
• Primary school	65	4.3	114	7.5
• Secondary school	506	33.3	812	53.4
• College / University	949	62.4	594	39.1
Income (SEK/month)				
• < 20,000	323	21.2	427	28.1
• 20,000–39,999	595	39.1	598	39.3
• ≥ 40,000	538	35.4	433	28.5
• Don’t know / Does not want to disclose	64	4.2	62	4.1
Living alone				
• Yes	366	24.1	378	24.8
• No	1154	75.9	1142	75.2
Health care region				
• Stockholm-Gotland	393	25.9	373.1	24.5
• Mid-Sweden	312	20.5	314.6	20.7
• South East	135	8.9	148.8	9.8
• South	253	16.6	277.1	18.2
• West	274	18.0	274.4	18.1
• North	153	10.1	131.9	8.7

Notes: SD, standard deviation; SEK, Swedish Krona. 10,000 SEK ≈ €885. ^a^ The unweighted mean (SD) age was 53.3 (17.5) years, while the weighted mean (SD) age was 48.7 (18.5) years

### Awareness of the ECAC

Overall, 3.7% of the respondents had heard about the ECAC before taking part in this survey. The number was highest among those aged 65–84 years old, where 6.1% had heard about the ECAC and lowest among those living alone (2.2%) (Table 3). In the weighted adjusted logistic regression analyses (Table 4), the results differed significantly for gender, education level, income, and cohabiting/living alone. Fewer males (OR 0.56; 95% CI 0.32–0.99; *P* = 0.048), those having an income of 20,000–39,999 SEK/month (compared with those in the group < 20,000 / Other, OR 0.49; 95% CI 0.24-1.00; *P* = 0.0496) and those living alone (OR 0.47; 95% CI 0.23–0.95; *P* = 0.035) had heard of the ECAC. Those having a college/university education were, however, 2.23 (95% CI 1.23–4.06) times more likely to have heard about the ECAC (*P* = 0.008).

**Table 3:** Awareness of ECAC according to the weighted distribution of the demographic characteristics

	Heard about ECAC before taking part in this survey^a^			Learned something new about cancer prevention^b^			Agreeing with the ECAC recommendations^c^		
Variable	*n*	%	*P*-value	*n*	%	*P*-value	*n*	%	*P*-value
Gender			**0.012**			0.212			**0.016**
• Male	18	2.4		465	60.6		437	56.9	
• Female	37	5.0		426	56.6		485	64.5	
Age (years)			0.129			**< 0.001**			**< 0.001**
• 18–34	11	2.5		338	79.0		299	70.0	
• 35–49	10	2.7		213	56.6		201	53.6	
• 50–64	13	3.7		181	50.0		190	52.5	
• 65–84	22	6.1		160	45.0		231	65.1	
National background			0.199			**0.042**			0.365
• Swedish	40	3.3		693	57.0		728	59.9	
• Other^d^	16	5.3		198	65.2		193	63.7	
College / University education			**0.023**			0.070			**0.019**
• Yes	32	5.3		367	61.8		385	58.0	
• No	24	2.6		524	56.6		537	64.8	
Income (SEK/month)			0.222			0.264			0.207
• < 20,000 / Other^e^	24	4.9		287	58.6		314	64.1	
• 20,000–39,999	17	2.8		335	56.0		361	60.5	
• ≥ 40,000	15	3.5		270	62.3		247	56.9	
Living alone			0.063			**0.015**			0.056
• Yes	8	2.2		196	52.0		209	55.4	
• No	47	4.1		695	60.8		712	62.4	
Health care region			0.774			0.137			0.915
• Stockholm-Gotland	10	2.6		194	52.0		223	59.8	
• Mid-Sweden	14	4.5		194	61.8		182	57.7	
• South East	5	3.5		83	55.9		94	63.0	
• South	9	3.2		175	63.0		173	62.4	
• West	12	4.2		159	58.0		171	62.4	
• North	6	4.8		86	64.9		79	60.0	

Notes: SEK, Swedish Krona. 10,000 SEK ≈ €885. Since frequencies and percentages are estimated based on post-stratification weights and then rounded, all numbers may not add up due to rounding errors. *P*-values are calculated using Pearson’s χ^2^-statistic with the Rao-Scott second-order correction. Significant *P*-values are given in **bold**. The overall weighted percentage of respondents answering “yes” (unweighted percentage of respondents answering “Don’t know”) was ^a^ 3.7% (1.2%), ^b^ 58.6% (2.6%), and ^c^ 60.6% (7.3%). ^d^ Including foreign and missing. ^e^ Including *Don’t know* and *Does not want to disclose*

On average, 58.6% responded that they had learnt something new about cancer prevention after being presented with the ECAC, with the highest results among respondents aged 18–34 years old (where 79% had learned something new). Respondents aged 65–84 years old reported the overall lowest number (where only 45% stated that they had learnt something new). In the adjusted regression analyses, we found a statistically significant (*P* < 0.001) age gradient, with fewer respondents reporting having learnt something new, OR (95% CI) 0.97 (0.96–0.98). Geographic area (HCR) was the only other variable with statistically significant differences, with those living in Mid-Sweden having a 1.53 (95% CI 1.05–2.25) times higher chance of having learnt something new (*P* = 0.028), while those living in the southern HCR had a 1.63 (95% CI 1.09–2.43) times higher chance (*P* = 0.017), compared to those living in Stockholm-Gotland.

In this sample, 60.6% of the respondents reported that they agreed with ECACs recommendations, with the highest overall agreement being observed among the younger respondents (18–34 years old). Here, 70.0% responded that they agreed with the recommendations. Agreement was lowest in the age group 50–64 years, where 52.5% stated that they agreed with the recommendations. However, in the adjusted regression analyses, education and income level, as well as living alone, were the only variables with statistically significant differences. Those with a college/university education were thus 1.48 (95% CI 1.14–1.91) times more likely to agree with the ECAC recommendations (*P* = 0.003), while those with an income of ≥ 40,000 SEK/month were less likely to agree with the recommendations, compared with those in the group < 20,000 / Other (OR 0.65; 95% CI 0.45–0.94; *P* = 0.023), as were those living alone (OR 0.73; 95% CI 0.54–0.98; *P* = 0.034).

**Table 4:** Results from weighted adjusted logistic regression analyses about awareness of ECAC

	Heard about ECAC before taking part in this survey			Learned something new about cancer prevention			Agreeing with the ECAC recommendations		
Variable	OR	95% CI	*P*-value	OR	95% CI	*P*-value	OR	95% CI	*P*-value
Male	0.56	0.32–0.99	**0.048**	1.16	0.89–1.52	0.269	0.80	0.61–1.06	0.115
Age (years)	1.02	1.00-1.04	0.052	0.97	0.96–0.98	**< 0.001**	1.00	0.99-1.00	0.226
Swedish background^a^	0.51	0.24–1.07	0.076	0.81	0.59–1.12	0.207	0.88	0.62–1.26	0.494
College / University education	2.23	1.23–4.06	**0.008**	1.30	1.00-1.69	0.050	1.48	1.14–1.91	**0.003**
Income (SEK/month)									
• < 20,000 / Other^b^	Ref.			Ref.			Ref.		
• 20,000–39,999	0.49	0.24-1.00	**0.0496**	0.94	0.68–1.29	0.685	0.84	0.59–1.18	0.314
• ≥ 40,000	0.58	0.28–1.19	0.140	1.11	0.78–1.57	0.576	0.65	0.45–0.94	**0.023**
Living alone	0.47	0.23–0.95	**0.035**	0.75	0.56–1.01	0.056	0.73	0.54–0.98	**0.034**
Health care region									
• Stockholm-Gotland	Ref.			Ref.			Ref.		
• Mid-Sweden	1.89	0.89–4.01	0.096	1.53	1.05–2.25	**0.028**	0.90	0.61–1.32	0.586
• South East	1.48	0.47–4.69	0.507	1.17	0.70–1.95	0.542	1.12	0.67–1.86	0.672
• South	1.28	0.56–2.92	0.555	1.63	1.09–2.43	**0.017**	1.09	0.72–1.64	0.690
• West	1.58	0.55–4.51	0.397	1.30	0.88–1.91	0.184	1.06	0.72–1.58	0.758
• North	2.15	0.83–5.55	0.113	1.55	0.95–2.53	0.081	0.96	0.60–1.52	0.848

Notes: CI, confidence interval; OR, odds ratio; Ref., reference category; SEK, Swedish Krona. 10,000 SEK ≈ €885. ^a^ Foreign background / Missing used as reference category. ^b^ Including *Don’t know* and *Does not want to disclose*

### Attitudes and behaviours related to lifestyle factors and cancer prevention

The results of the three variables measuring attitudes, behaviours related to lifestyle factors and cancer prevention (according to the weighted distribution of the demographic characteristics) are presented in Table 5. Overall, 27.4% of the respondents reported that their motivation to improve their lifestyle increased after reading the ECAC, with the highest and lowest percentages found in the age groups 18–34 (35.6%) and 50–64 (22.0%) years old, respectively. In the weighted adjusted logistic regression analyses (Table 6), statistically significant differences were only found for age and education levels. Amongst the older respondents, fewer reported motivation to change (OR 0.99; 95% CI 0.98-1.00; *P* = 0.009). Those with a college/university education, however, had a 1.34 (95% CI 1.01–1.78; *P* = 0.0042) times higher chance of reporting increased motivation.

**Table 5:** Attitudes and behaviours related to lifestyle factors and cancer according to the weighted distribution of the demographic characteristics

	Motivation to improve lifestyle increased after reading the ECAC^a^			Have changed lifestyle after being informed about cancer prevention^b^			Intends to change lifestyle to decrease the risk of cancer^c^		
Variable	*n*	%	*P*-value	*n*	%	*P*-value	*n*	%	*P*-value
Gender			0.110			**0.011**			**0.017**
• Male	192	25.1		105	13.6		197	25.7	
• Female	224	29.8		143	19.0		246	32.7	
Age (years)			**0.003**			0.070			0.081
• 18–34	152	35.6		54	12.6		148	34.7	
• 35–49	92	24.4		71	19.0		93	24.8	
• 50–64	79	22.0		53	14.8		105	29.1	
• 65–84	93	26.2		69	19.5		97	27.4	
National background			0.697			0.139			**0.040**
• Swedish	329	27.1		187	15.4		335	27.5	
• Other^d^	87	28.6		61	20.0		109	35.8	
College / University education			0.081			**< 0.001**			0.356
• Yes	180	30.4		137	23.0		183	30.8	
• No	236	25.5		111	12.0		261	28.2	
Income (SEK/month)			0.220			0.396			0.571
• < 20,000 / Other^e^	150	30.7		75	15.3		153	31.4	
• 20,000–39,999	148	24.8		92	15.4		170	28.5	
• ≥ 40,000	118	27.2		81	18.7		120	27.7	
Living alone			0.372			0.517			0.207
• Yes	95	25.2		66	17.6		98	26.0	
• No	321	28.1		181	15.9		346	30.3	
Health care region			0.826			0.560			0.157
• Stockholm-Gotland	106	28.3		68	18.1		96	25.8	
• Mid-Sweden	80	25.5		57	18.0		99	31.5	
• South East	36	24.3		21	14.0		47	31.7	
• South	81	29.2		39	14.2		68	24.5	
• West	71	26.0		47	17.3		81	29.6	
• North	42	31.7		16	12.0		52	39.3	

Notes: SEK, Swedish Krona. 10,000 SEK ≈ €885. Since frequencies and percentages are estimated based on post-stratification weights and then rounded, all numbers may not add up due to rounding errors. *P*-values are calculated using Pearson’s χ^2^-statistic with the Rao-Scott second-order correction. Significant *P*-values are given in **bold**. The overall weighted percentage of respondents answering “yes” (unweighted percentage of respondents answering “Don’t know”) was ^a^ 27.4% (4.2%), ^b^ 16.3% (5.3%), and ^c^ 29.2% (4.6%). ^d^ Including foreign and missing. ^e^ Including *Don’t know* and *Does not want to disclose*

For the outcome ”Having changed lifestyle after being informed about cancer prevention”, on average 16.3% of the respondents reported as having changed their lifestyles. The highest percentage (23.0%) was found among respondents with a college/university education, while the lowest percentage (12.0%), was found among those without a college/university education, as well as those living in the north of Sweden (north HCR). In the adjusted regression analyses, statistically significant differences were found for gender (*P* = 0.044) and education levels (*P* < 0.001). Here, males were less likely (OR 0.72; 95% CI 0.53–0.99) and those with a college/university education more likely (OR 2.1; 95% CI 1.50–2.98) to report having changed their lifestyle.

On average, 29.2% of the respondents reported having intentions of changing their lifestyle, in order to decrease their risk for cancer. The highest percentage was found among those living in the northern HCR, where 39.3% reported intentions of changing their lifestyle, while the lowest percentage (24.5%) was found among those living in the southern HCR. In the adjusted regression analyses, statistically significant differences were found for gender, national background, and HCR. Here, males (OR 0.74; 95% CI 0.55–0.99; *P* = 0.041) and those with a Swedish background (OR 0.68; 95% CI 0.48–0.99; *P* = 0.041) were less likely to respond that they intended to change their lifestyles. On the contrary, those living in the northern HCR were 1.82 (95% CI 1.12–2.96; *P* = 0.015) times as likely to respond that they intended to change their lifestyle, compared to those living in the Stockholm-Gotland HCR.

**Table 6:** Results from weighted adjusted logistic regression analyses about attitudes and behaviours related to lifestyle factors and cancer

	Motivation to improve lifestyle increased after reading the ECAC			Have changed lifestyle after being informed about cancer prevention			Intends to change lifestyle to decrease the risk of cancer		
Variable	OR	95% CI	*P*-value	OR	95% CI	*P*-value	OR	95% CI	*P*-value
Male	0.82	0.60–1.11	0.195	0.72	0.53–0.99	**0.044**	0.74	0.55–0.99	**0.041**
Age (years)	0.99	0.98-1.00	**0.009**	1.01	1.00-1.02	0.071	0.99	0.99-1.00	0.221
Swedish background^a^	1.01	0.69–1.48	0.968	0.69	0.46–1.05	0.084	0.68	0.48–0.99	**0.041**
College / University education	1.34	1.01–1.78	**0.042**	2.11	1.50–2.98	**< 0.001**	1.17	0.88–1.54	0.278
Income (SEK/month)									
• < 20,000 / Other^b^	Ref.			Ref.			Ref.		
• 20,000–39,999	0.75	0.52–1.08	0.122	0.90	0.60–1.35	0.602	0.89	0.63–1.27	0.524
• ≥ 40,000	0.80	0.54–1.17	0.246	1.00	0.66–1.51	0.988	0.87	0.59–1.28	0.483
Living alone	0.85	0.60–1.20	0.355	1.12	0.77–1.64	0.556	0.82	0.59–1.13	0.220
Health care region									
• Stockholm-Gotland	Ref.			Ref.			Ref.		
• Mid-Sweden	0.87	0.57–1.32	0.501	1.09	0.70–1.69	0.704	1.32	0.88–1.98	0.179
• South East	0.78	0.46–1.35	0.380	0.86	0.48–1.55	0.617	1.34	0.79–2.28	0.272
• South	1.04	0.66–1.65	0.865	0.80	0.47–1.34	0.396	0.92	0.59–1.43	0.703
• West	0.86	0.55–1.33	0.490	0.97	0.60–1.55	0.889	1.17	0.76–1.80	0.477
• North	1.10	0.66–1.85	0.714	0.67	0.37–1.22	0.192	1.82	1.12–2.96	**0.015**

Notes: CI, confidence interval; OR, odds ratio; Ref., reference category; SEK, Swedish Krona. 10,000 SEK ≈ €885. ^a^ Foreign background / Missing used as reference category. ^b^ Including *Don’t know* and *Does not want to disclose*

## Discussion

To our knowledge, this is the first study examining the awareness and attitudes towards the ECAC among the general public in Sweden. The results from this cross-sectional study show that a very small proportion (4%) of the respondents had heard about the ECAC before participating in this study. After having been presented with the ECAC, just over half of the respondents stated that they learned something new about cancer prevention, and three out of five agreed with the recommendations presented in the code. A smaller portion (27%) agreed that reading the ECAC recommendations motivated them to improve their lifestyle to reduce their cancer risk.

Results in context.

Previous studies examining ECAC awareness have come to varying conclusions. One study, polling 8171 people from eight European countries, found that awareness of the ECAC ranged from 2% in the United Kingdom (UK) to 21% in Hungary and Poland [23]. A Spanish study found that 13.3% amongst the general public [28] and 23.3% among medicine and nursing students [29] were aware of the ECAC. Evidently, the goal set in Europe’s Beating Cancer Plan, to reach an 80% awareness level of the ECAC among the European population in 2025 [3], is far from achieved. In Sweden, the ECAC has mainly been promoted by the Regional Cancer Centres (focusing primarily on health care professionals and people with a cancer diagnosis) and affiliated actors [30, 31]. This could explain the low awareness levels in the general population.

Information on cancer prevention can come from a breadth of other sources than the ECAC. In Sweden, cancer prevention communication is provided in numerous formats by various actors, such as national authorities, health care providers, The Swedish Cancer Society, and patient organisations. It is important for the European Commission, the EU members states, national authorities, and public health actors to discuss the aims of the ECAC going forward. Is it intrinsically valuable for the general public in Europe to know about cancer prevention through the ECAC? Or should the focus rather be on improving cancer prevention knowledge through a multitude of efforts and channels, including the ECAC?

Over half of the participants in this study stated that they learned something new after reading the ECAC, indicating a need to improve the general public’s awareness of cancer preventive measures. Notably, this figure was highest in the youngest age group (18–34 years). In a previous study investigating if the ECAC adds new knowledge regarding cancer prevention, Ritchie et al. [23] report significant variation between eight European countries, ranging from 39% of respondents learning something new from the code in the UK, to 80% in Poland. Moreover, in their sample, between 38% (UK) and 88% (Portugal) of respondents claimed that they would likely make changes to their lifestyle as a result of reading the ECAC. In our study, the corresponding figure was 27.4%. Notably, motivation to improve lifestyle increased with education level and decreased with older age. We also found that less than one in five stated that previously received information on cancer prevention had made them improve their lifestyle.

Data from the Swedish Public Health Agency show that over half of the Swedish adult population is overweight or obese, 35% of adults are not reaching the recommended levels of physical activity, 15.5% of adults consume alcohol above the levels classified as risky drinking, and only 17.2% of adults consume fruits and vegetables more than three times a day [32]. This indicate that a greater proportion of people found in this study probably have reasons to improve their lifestyles. These results need to be seen in the light of health behaviour theories, as many factors other than inner motivation affect behaviour. Perceived benefits, susceptibility, and barriers, as well as societal norms and attitudes, are among other factors that can shape behaviours [33, 34]. Furthermore, social desirability bias might influence survey respondents when answering questions regarding health and lifestyle [35].

It has previously been described that to improve public health, interventions addressing socioeconomic determinants of health have the largest impact, while counselling and information are often less effective and require more individual effort [36]. However, even though information on risk factors and recommendations alone are not sufficient to change people’s behaviours, being well informed is a prerequisite to engage in cancer prevention activities [37]. The content of the ECAC [8] and information on cancer prevention in general [38] have previously been described as overwhelming and difficult to fully comprehend. Although not an aim of this study, this highlights the importance of considering target group health literacy (HL) when producing and distributing health information material. HL is defined as the ability of individuals to gain access to, understand, use, and critically assess information in ways that promote and maintain good health for themselves, their families, and their communities [39]. Low HL has been suggested to be associated with lower perceived control over cancer risk factors [40] and longer primary care intervals (i.e. number of days from patients’ initial presentation of symptoms in primary care until referral to further examinations) [41].

Previous research has indicated that information concerning cancer prevention needs to be enhanced as well as tailored to target specific populations, in order to minimize the risk of misconceptions and stigmatization [2, 8]. HL is thus not primarily an individual responsibility. Governments and public health organizations have a responsibility to ensure that accurate, appropriate, and accessible information is provided. Information that underpins well-informed decisions regarding health should be tailored to meet the needs of a broad and diverse population [39, 42, 43]. The ECAC has a large and diverse target audience and its format of cancer prevention messaging may not meet the needs of this broad and diverse population in a satisfactory manner. This highlights the need for continued efforts to enhance cancer prevention communication, tailored for different population groups, as well as for societies to utilize a wide range of interventions to reduce the cancer burden in Europe. Population-based public health information is one important cancer prevention strategy that needs to be applied in tandem with other efforts.

The results from the present study add important insights to the current (yet scarce) literature on awareness and attitudes towards the ECAC. However, more research is required regarding how the ECAC is perceived and received by the target audiences, i.e., the European general population. This is especially relevant given the results of this study, indicating that the ECAC does not increase most peoples’ motivation to improve their lifestyles. The low ECAC awareness and its limited ability to increase motivation towards a healthier lifestyle found in this study and elsewhere [23, 24, 28, 29], is a call for consideration on how this tool should be utilized to produce higher dividends to the European population. The fact that an updated version of the ECAC is to be launched and communicated during 2025 and onwards makes this discussion particularly urgent.

### Strengths and limitations

Among the strengths of this study was the use of a large representative sample of the general Swedish population as well as using post-stratification weights in the statistical analyses, making the results even more representative for the general public. With Sweden being amongst the leading countries regarding utilization of digital technologies, there are comparably small differences in uptake between population groups [44]. Although the Sweden Panel is representative of the Swedish general population in terms of age, sex, and geographical region, one limitation was the inability for non-Swedish speaking persons and people without digital access to complete the survey, which may have resulted in some selection bias. A further limitation of using the Sweden Panel was that the researchers did not have control of the data collection process and e.g. how the post-stratification and weighting were implemented.

The observational cross-sectional design was considered suitable to answer the research questions. Yet, when reviewing the results, it is important to consider the customary shortcomings of a cross-sectional study design [45]. However, when repeated, studies of this kind are a feasible way of tracking trends in cancer prevention awareness over time. In a Swedish context, the results from this study can be used to shape and prioritize public health interventions in the coming years. From an international point of view, the results can serve as a point of comparison for other countries, as well as inform researchers, decision makers, and public health institutions. A final limitation was that the questionnaire used in the study was not validated. It was, however, developed in collaboration between the research group and the survey experts at Novus to facilitate comparison with previous research, which should be considered a strength.

## Conclusion

Awareness of the ECAC among the general public in Sweden is very low. Still, a majority seem to agree with its recommendations. The results of the present study also indicate that the ECAC motivates some, but far from all, to improve their lifestyle habits in order to reduce their cancer risk. Consequently, further research is warranted on how the ECAC best could and should be used in order to improve cancer prevention awareness and motivation.

## Electronic supplementary material

Below is the link to the electronic supplementary material.

Supplementary Material 1

## Data Availability

The datasets used and analysed during the current study are available from the corresponding author, on reasonable request.

## Abbreviations

CI: Confidence interval
ECAC: European code against cancer
EU: European Union
HCR: Health care regions
HL: Health literacy
OR: Odds ratio
SD: Standard deviation
WHO: World Health Organization
