# Supplementary material for: Low levels of awareness and motivation towards cancer prevention amongst the general public in Sweden: a cross-sectional study focusing on the European Code Against Cancer
Source: BMC Public Health. 2025 May 7;25:1692. doi: 10.1186/s12889-025-22803-3 (PMC12057043; doi:10.1186/s12889-025-22803-3)
Supplement: Supplementary file 1 — Supplementary Material 1 [file 12889_2025_22803_MOESM1_ESM.docx]

**Questionnaire**

***To what extent do you agree with the following statement?***

1. **I believe that people can reduce their risk of getting cancer by changing their lifestyle habits**

Scale: 1-5 Totally agree – disagree completely /Do not know

1. **What proportion of cancer cases in Sweden do you think is due to unhealthy lifestyle habits?**
   1. None
   2. 1 out of 10
   3. 2 out of 10
   4. 3 out of 10
   5. 4 out of 10
   6. 5 out of 10
   7. 6 out of 10
   8. 7 out of 10
   9. 8 out of 10
   10. 9 out of 10
   11. All
   12. Do not know
2. **Would you say that the following factors increase the risk of developing cancer?**
   1. smoking
   2. heredity
   3. solar radiation
   4. sunbeds
   5. air pollution
   6. radon
   7. secondhand smoking
   8. alcohol
   9. snus
   10. obesity
   11. overweight
   12. hormone therapy
   13. burnt food
   14. processed meat (e.g. bacon, sausages, ham)
   15. low level of physical activity
   16. red meat
   17. low intake of fruit and vegetables (<500g /day)
   18. low fiber intake
   19. viral and bacterial infections
   20. additives in food
   21. stress
   22. e-cigarettes
   23. sedentary
   24. food rich in fat
   25. low intake of whole grains
   26. too little sleep
   27. sugar
   28. artificial sweeteners
   29. not breastfeeding your child
   30. coffee
   31. milk
   32. using mobile phones

*Scale: Yes/No/Do not know*

***Now some questions about cancer prevention, which means different ways to prevent cancer and/or reduce the cancer risk***

**To what extent do you agree with the following statements?**

1. **It is too late for me to change my lifestyle to reduce my risk for getting cancer**
2. **I am interested in cancer prevention information**
3. **There is too much information about cancer prevention**Scale 1-5: Totally agree – disagree completely /Do not know

***Below is the European Code Against Cancer. Read it through and then press the Next button.***

**
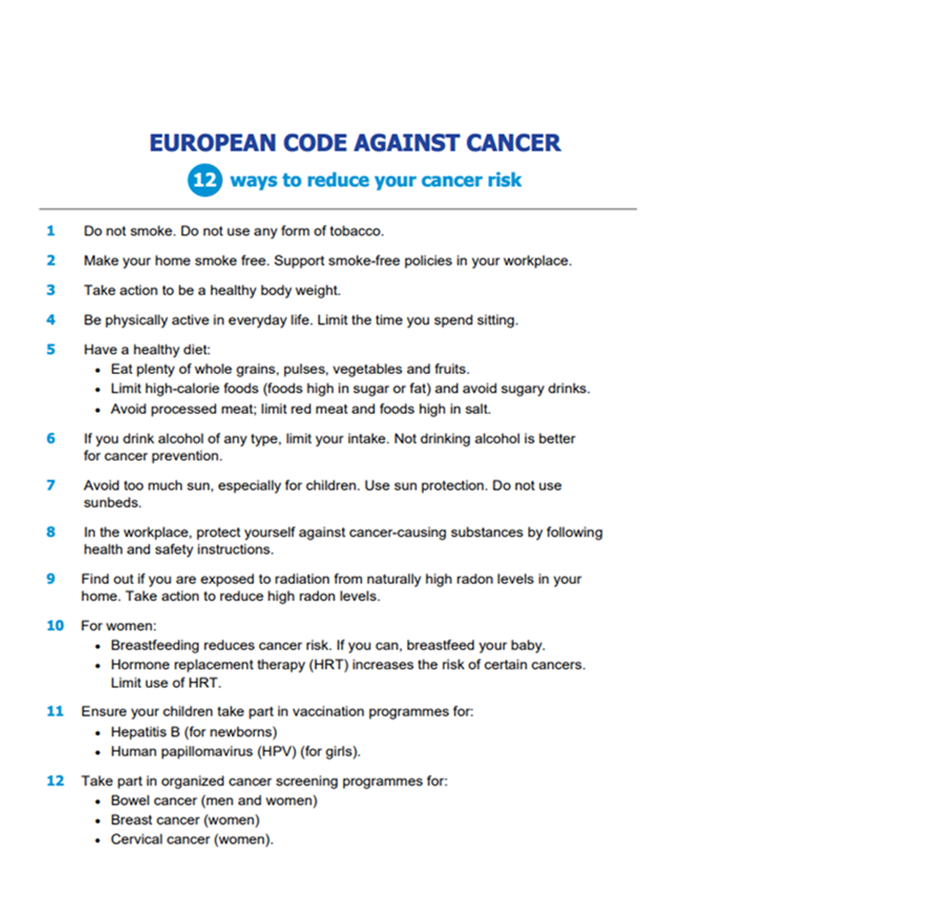
** ***https://cancer-code-europe.iarc.fr/index.php/en/***

1. **Had you heard of the European Code Against Cancer before taking part in this survey?**

*Scale: Yes/No/ Do not know*

**If yes question 7:**

1. **How well do you know the European Code Against Cancer?**

Scale: Know very well/Know pretty well/ Know little/Heard about but hardly know anything

***To what extent do you agree with the following statements?***

1. **I have learned something new about cancer prevention after reading the European Code Against Cancer**
2. **I agree with the recommendations described in the European Code Against Cancer**
3. **My motivation to improve my lifestyle habits has increased after reading the European Code Against Cancer**
4. **Information about cancer prevention has made me change my lifestyle**
5. **I intend to change my lifestyle to reduce my risk of cancer.**
6. **I would like to know if I have a higher risk of getting cancer due to genetics/heredity**

*Scale: 1-5 Totally agree – disagree completely /Do not know*

1. **Are you trying to do any of the following:**
   1. reduce the number of cigarettes you smoke
   2. stop smoking
   3. increase intake of fruit and vegetables
   4. decrease intake of processed meat (eg. bacon, sausages, salami)
   5. be more physically active
   6. reduce intake of alcohol
   7. reduce body weight

*Scale: Yes/ No/ Prefer not to answer/ Not applicable to me*
